# Multitarget Tracking Algorithm Using Multiple GMPHD Filter Data Fusion for Sonar Networks

**DOI:** 10.3390/s18103193

**Published:** 2018-09-21

**Authors:** Xueli Sheng, Yang Chen, Longxiang Guo, Jingwei Yin, Xiao Han

**Affiliations:** 1 Acoustic Science and Technology Laboratory, Harbin Engineering University, Harbin 150001, China; shengxueli@hrbeu.edu.cn (X.S.); cy5311@hrbeu.edu.cn (Y.C.); yinjingwei@hrbeu.edu.cn (J.Y.); hanxiao1322@hrbeu.edu.cn (X.H.); 2 Key Laboratory of Marine Information Acquisition and Security (Harbin Engineering University), Ministry of Industry and Information Technology, Harbin 150001, China; 3 College of Underwater Acoustic Engineering, Harbin Engineering University, Harbin 150001, China

**Keywords:** multisensor data fusion, multitarget tracking, GMPHD, sonar network, RFS

## Abstract

Multitarget tracking algorithms based on sonar usually run into detection uncertainty, complex channel and more clutters, which cause lower detection probability, single sonar sensors failing to measure when the target is in an acoustic shadow zone, and computational bottlenecks. This paper proposes a novel tracking algorithm based on multisensor data fusion to solve the above problems. Firstly, under more clutters and lower detection probability condition, a Gaussian Mixture Probability Hypothesis Density (GMPHD) filter with computational advantages was used to get local estimations. Secondly, this paper provided a maximum-detection capability multitarget track fusion algorithm to deal with the problems caused by low detection probability and the target being in acoustic shadow zones. Lastly, a novel feedback algorithm was proposed to improve the GMPHD filter tracking performance, which fed the global estimations as a random finite set (RFS). In the end, the statistical characteristics of OSPA were used as evaluation criteria in Monte Carlo simulations, which showed this algorithm’s performance against those sonar tracking problems. When the detection probability is 0.7, compared with the GMPHD filter, the OSPA mean of two sensor and three sensor fusion was decrease almost by 40% and 55%, respectively. Moreover, this algorithm successfully tracks targets in acoustic shadow zones.

## 1. Introduction

The issue of multiple target tracking (MTT) has emerged as an area of interest in radar, sonar, etc. Traditionally, there are many classical MTT algorithms based on explicit data association information, such as probability data association (PDA) [1,2], joint probability data association (JPDA) [3,4,5], multiple hypothesis tracking (MHT) [6] and derivative algorithms [7,8]. As the key of these MTT algorithms is data association, the data association algorithm usually causes computational bottlenecks when the number of targets is too large. Therefore, these algorithms usually perform poorly when the number of targets is large.

In response, the random finite set (RFS) [9,10] has attracted the attention of scholars engaged in MTT algorithm research. As no explicit data association is required, MTT algorithms based on RFS have a computational advantage [11,12]. In the last 15 years, the probability hypothesis density (PHD) [10], cardinalized PHD (CPHD) filter [13], sequential Monte Carlo PHD (SMCPHD) [14], Gaussian Mixture PHD (GMPHD) [15] and multi-Bernoulli filters [16] have been proposed for MTT. In 2013, the notion of labeled RFS [17] was introduced to address target trajectories and their uniqueness. Thus, by utilizing the labeled RFS theory, the labeled multi-Bernoulli (LMB) filter [18,19] and generalized labeled multi-Bernoulli (GLMB) [20] filter have advantages in target track estimation and low signal to noise ratio (SNR). Vo proposed an efficient implementation of the GLMB filter based on Gibbs sampling, which has linear complexity in the number of measurements, but at least quadratic in the number of targets [21].

In recent years, people have increased the research and development of ocean resources, so the collection of ocean information has consequently become important. In addition, considering the unpredictable dangers of underwater and harsh working conditions, a growing number of buoy sonar and underwater unmanned vehicles (UUVs) are responsible for underwater information collection. Since these sonar devices are powered by batteries and transmit the preprocessing results of collected information periodically to communication buoys, efficient information processing is particularly important. Moreover, more clutters, poor detection accuracy and complex channels all make sonar detection difficult, weakening the accuracy of MTT. 

Unluckily, the PHD filter is designed for high SNR, while the sonar work environments are lower SNR. Distributed multisensor data fusion not only compensates for the lack of information caused by low SNR, but also improves the tracking accuracy [22,23,24]. Distributed fusion architectures composed of tracker, data association and fusion are characterized by low communication bandwidth demands, high system reliability and strong survivability. On the other hand, distributed sensor networks also have another advantage in detection coverage (e.g., acoustic shadow zones).

The purpose of this paper is to propose an efficient MTT algorithm for sonar detection systems. The structure of this paper is as follows: in the Section 2, we analyze the problems of sonar detection systems. Section 3 presents the classical GMPHD filter algorithm. The maximum-detection capability multitarget track fusion (MDC-MTF) algorithm is proposed in Section 4, and Monte Carlo simulations are provided in Section 5. In the Section 6, the conclusions are presented.

## 2. Problem Analysis and Solutions

In order to make sure this algorithm could successfully solve above multitarget problems based on sonar sensors, this algorithm framework and three analyses are provided in this section.

### 2.1. Computational Bottle-Neck

Many papers [11,12,18,19,20] have analyzed in depth the computational complexity of PHD filter and others MTT algorithms. The explicit data association-based algorithms (e.g., MHT, JPDA) suffer from prohibitive computational complexity with increasing number of targets and measurements. For example, the amount of computation will increase exponentially with the increase of the number of targets. However, without explicit data association algorithms, the PHD filter has a linear computational complexity O(mn), where m is the number of detections and n is number of targets. Hence, PHD filter can solve the computational bottleneck problem better.

### 2.2. Lower Probability Detection and Acoustic Shadow Zone

**Figure 1:**
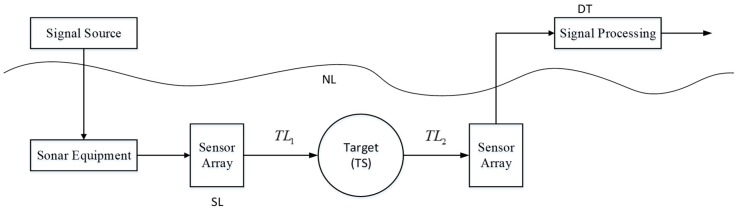
A simple schematic of active sonar detection.

It is well known that sonar is always working with noise. A simple active sonar detection schematic is shown in Figure 1. A detecting signal is emitted by a sonar sensor array with sound level *SL*. After the transmission loss of *TL*_1_, the signal reaches the target. When the target’s scattering strength is *TS*, the sound level of the scattering signal is *SL* − *TL*_1_ + *TL*. After the transmission loss of *TL*_2_ the signal is received by the receiver sonar sensor array. Let the receiver noise level be denoted as *NL*, and *DI* denotes the receiver directivity index. When the received signal of sound level is not less than the detection threshold *DT*, the target can be detected:

(1) $$SL-TL_{1}-TL_{2}+TS-\left(NL-DI\right)\geq DT$$

**Figure 2:**
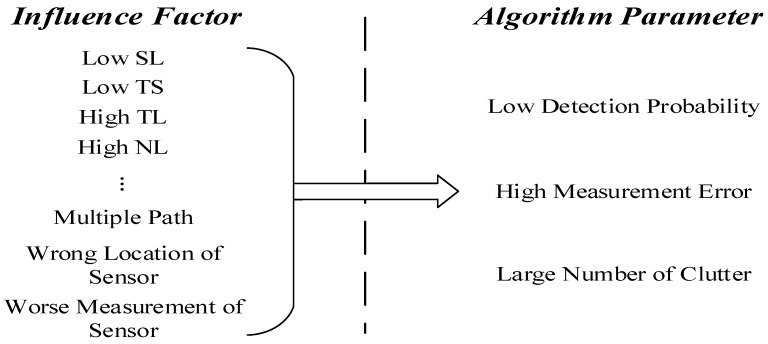
The relationship between influence factors and algorithm parameters.

In the Figure 2, we can see that there are many factors influencing target detection. However, these influences can be reflected by detection probability, number of clutters and measurement errors in the MTT algorithm.

**Figure 3:**
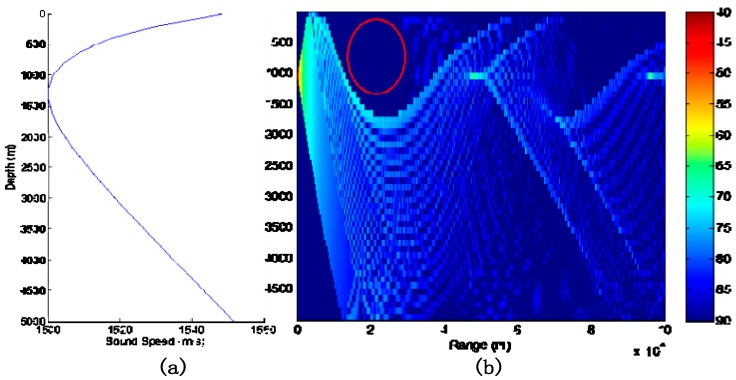
Example of sound acoustic zone: (**a**) the speed of sound; (**b**) the transmission loss, where the red circle is an acoustic shadow zone.

For example, as shown in Figure 3, when a target is located in the acoustic shadow zone (red circle) of the sensor it can be deemed that the transmission loss of the target is very large. Thus, the detection probability of the target is very small, which results in little effective measurement data for the target. This is also the reason for studying the MTT algorithm for low SNR situations.

Luckily, distributed multiple sensor networks have a huge advantage in MTT by fusing multisensor data. For example, although a target is located in the acoustic shadow zone of sensor 1, the MTT algorithm can still track the target when it can be detected by sensor 2. In addition, when a target can be detected by multiple sensors at the same time, the MTT algorithm can achieve statistical accuracy improvement.

### 2.3. Framework of Maximum-Detection Capability Multitarget Track Fusion Algorithm

Distributed fusion structure is a common fusion method in which each local sensor has a tracker, and the local track calculated from the tracker is sent to the fusion center. In the fusion center, all the tracks will be associated and fused to estimate global tracks. The distributed fusion structure has the following advantages: low communication burden, high reliability, easy implementation and computational balance. Moreover, MTT algorithms based on distributed fusion structure have the capability of local tracking and global monitoring. In sonar detection networks, MTT algorithms based on a distributed fusion structure could also track targets in acoustic shadow zones.

**Figure 4:**
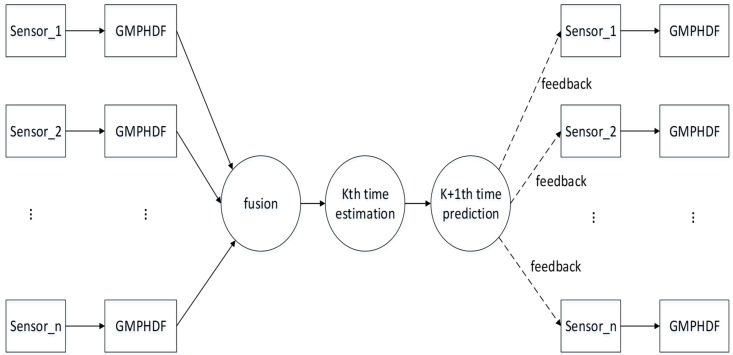
Framework of the maximum-detection capability multitarget track algorithm.

Therefore, this paper proposes a MDC-MTF algorithm. As Figure 4 shows, the GMPHD filter is firstly used to get a local estimation from local sonar sensor measurements. Secondly, association and fusion algorithms are used to estimate the global tracking result. Thirdly, a novel feedback algorithm is used to improve the local sensor tracking performance.

## 3. GMPHD Filter Theory

In a multitarget tracking environment, target states finite sets *X_k_* and measurements finite sets *Z_k_* are determined as follows:

(2) $$X_{k}=\left\{x_{k,1},x_{k,2},\cdots ,x_{k,N\left(k\right)}\right\}$$

(3) $$Z_{k}=\left\{z_{k,1},z_{k,2},\cdots ,z_{k,M\left(k\right)}\right\}$$

where *M*(*k*) and *N*(*k*) are respective number of targets state *x_k_*_,1_, …, *x_k,M_*_(*k*)_ ∈ *X_k_* and measurements *z_k_*_,1_, …, *z_k,N_*_(*k*)_ ∈ *Z_k_* at time *k*.

For a given multitarget state *X_k_*_−1_ at time *k* − 1, each *x_k_*_−1_ ∈ *X_k_*_−1_ either continues to exist at time *k* with probability *P_S,k_*(*x_k_*_−1_), or dies with probability 1 − *P_S,k_*(*x_k_*_−1_). Hence this behavior could be modelled as a RFS $S_{k|k-1}\left(x_{k-1}\right)$. At time *k*, a new target can arise by spontaneous birth or by spawning from an exist target at time *k* − 1. Also, they could be modelled spontaneous births sets *Γ_k_* and spawned target sets $B_{k|k-1}\left(x_{k-1}\right)$ as a RFS at time *k*. Therefore, at time *k*, a given multitarget state *X_k_* consists of three sets of $S_{k|k-1}\left(Ϛ\right)$, $B_{k|k-1}\left(Ϛ\right)$ and *Γ_k_*:

(4) $$X_{k}=\left[\underset{Ϛ\in X_{k-1}}{⋃}S_{k|k-1}\left(Ϛ\right)\right]⋃[\underset{Ϛ\in X_{k-1}}{⋃}B_{k|k-1}\left(Ϛ\right)]⋃\Gamma_{k}$$

Moreover, at time *k*, each target could be detected by sensor with probability $P_{D,k}\left(x_{k}\right)$. Each target state $x_{k}\in X_{k}$ could generate a RFS $\Theta_{k}\left(x_{k}\right)$ at time *k*. In addition, the sensor also could receive some false measurements or clutter at time *k*. They can be modelled as a RFS $K_{k}$. Consequently, the RFS measurement set $Z_{k}$ can be described as follows:

(5) $$Z_{k}=K_{k}⋃\left[\underset{x\in X_{k}}{⋃}\Theta_{k}\left(x\right)\right]$$

Let $p_{k}(•|Z_{1:k})$ denote the multitarget posterior density, $f_{k|k-1}(•|•)$ denote the multitarget transition density, and $g_{k}(•|•)$ denote the multitarget likelihood. Then, based on optimal multitarget Bayes filter theory, the multitarget posterior can be propagated by the recursion:

(6) $$p_{k|k-1}\left(X_{k}|Z_{1:k-1}\right)=\int f_{k|k-1}(X_{k}|X)p_{k-1}(X|Z_{1:k-1})\mu_{s}\left(dX\right)$$

(7) $$p_{k}\left(X_{k}|Z_{1:k}\right)=\frac{g_{k}(Z_{k}|X_{k})p_{k|k-1}(X_{k}|Z_{1:k-1})}{\int g_{k}\left(Z_{k}|X\right)p_{k|k-1}(X|Z_{1:k-1})\mu_{s}\left(dX\right)}$$

where $\mu_{s}$ is an appropriate reference measure on *F*(*χ*) [14].

We assume that each target evolves and generates observations independently of one another, the clutter is independent of target-originated measurements, and the clutter and predicted multitarget RFS follow a Poisson distribution. Then, let $v_{k}\left(•\right)$ denote the multitarget posterior density intensity, the posterior intensity can be propagated by the PHD recursion:

(8) $$v_{k|k-1}\left(x\right)=\int P_{S,k}\left(Ϛ\right)f_{k|k-1}\left(x|Ϛ\right)v_{k-1}\left(Ϛ\right)dϚ+\int \beta_{k|k-1}\left(x|Ϛ\right)v_{k-1}\left(Ϛ\right)dϚ+\gamma_{k}\left(x\right)$$

(9) $$v_{k}\left(x\right)=\left[1-P_{D,k}\left(x\right)\right]v_{k|k-1}\left(x\right)+\underset{z\in Z_{k}}{\sum }\frac{P_{D,k}\left(x\right)g_{k}(z|x)v_{k|k-1}\left(x\right)}{\kappa_{k}\left(z\right)+\int P_{D,k}\left(\xi \right)g_{k}(z|\xi )v_{k|k-1\left(\xi \right)d\xi }}$$

According to Gaussian mixture model (GMM) theory and GMPHD algorithm [15], the Equations (10) and (11) could be substituted by Equations (8) and (9):

(10) $$v_{k-1}\left(x\right)=\overset{J_{k-1}}{\underset{i=1}{\sum }}\omega_{k-1}^{\left(i\right)}N\left(x;m_{k-1}^{\left(i\right)},P_{k-1}^{\left(i\right)}\right)$$

(11) $$v_{k|k-1}\left(x\right)=\overset{J_{k|k-1}}{\underset{i=1}{\sum }}\omega_{k|k-1}^{\left(i\right)}N\left(x;m_{k|k-1}^{\left(i\right)},P_{k|k-1}^{\left(i\right)}\right)$$

where, ω is the weight of Gaussian distribution, N(•;m,P) denotes a Gaussian density with mean *m* and covariance *P*, *J* is the number of components of the intensity. Therefore, the prediction updating and estimation of the target can be implemented. For the implementation process, please refer to the paper [15].

## 4. The MDC-MTF Algorithm

In practical applications, sonar equipment usually only acquires the target location information, MTT algorithms need to start tracking according to the target location information, while the target speed information is essential for most MTT Bayes trackers. However, these track initiation algorithms are sensitive to SNR. The performance of tracker may be severely degraded when the SNR decreases. Thus, a MDC-MTF algorithm and a novel feedback algorithm were proposed in this paper to improve the GMPHD filter performance.

### 4.1. Maximum Detection Capability Fusion Strategy

In the distributed fusion structure, most of the clutter has been filtered out by GMPHD filter. Then, all local estimations will be associated and fused at a fusion center. In order to ensure the maximum detection capability, we divide the local estimates into two categories. One is the correlated local estimates, and the other is the uncorrelated local estimates. For the correlated local estimates, we associate and fuse those local estimates. For the uncorrelated local estimates, we treat them as a global estimation. Usually, there are three cases in associating two local estimates:

Case 1: the local estimation from sensor *i* can match with sensor *j*;
Case 2: the local estimation from sensor *i* mismatch with sensor *j*;
Case 3: the target state does not exist in sensor *i* and sensor *j*;

Therefore, this paper proposes a fusion strategy:

(a) For case 1, the local estimation from two sensors association, and the most possible data fusion to estimate target state;
(b) For case 2, the local estimation is retained as a global estimation;
(c) For case 3, treated them as missing detection.

### 4.2. Data Association Algorithm

Unlike the JPDA algorithm, the target density has been significantly reduced after GMPHD filtering, so the association algorithm does not result in a heavier computational burden in MDC-MTF. Today, there are many classical data association algorithms, such as nearest neighbor (NN) [25], weighted track association (WTA) [26], modified weighted track association (m-WTA) [27], k-nearest neighbor (k-NN), modified k-nearest neighbor (MK-NN) [28], independent and dependent sequential track correlation criteria (STCC), independent and dependent binary track correlation (BTC) algorithms, and fuzzy synthetic track correlation criterion (FSTCC). A detailed analysis of association performance was presented in the paper [29]. The performance comparison of track correlation algorithms is shown in Table 1.

As shown in Table 1, the weighted track association algorithm is an optimal choice considering the computational cost, correct correlation probability (medium target density) and communication burden. The weighted track association algorithm is described as follows:

At time *k*, $t_{gh}\left(k\right)$ is defined as the difference in the value of two sensors:

(12) $$t_{gh}\left(k\right)={\hat{X}}_{g}^{i}\left(k\right)-{\hat{X}}_{h}^{j}\left(k\right)$$

where ${\hat{X}}_{g}^{i}$ and ${\hat{X}}_{h}^{j}$ were respectively the *g*-th local estimation of sensor *i* and *j* the *h*-th local estimation of sensor. Also, $P_{g}^{i}$ and $P_{h}^{j}$ are defined as covariance. When the errors of sensors are uncorrelated, the Mahalanobis distance $\alpha_{gh}$ could be calculated by Equations (13) and (14):

(13) $$\alpha_{gh}\left(k\right)=t_{gh}^{\prime }\left(k\right)C_{gh}{\left(k\right)}^{-1}t_{gh}\left(k\right)$$

(14) $$C_{gh}\left(k\right)=P_{g}^{i}\left(k\right)+P_{h}^{j}\left(k\right)$$

According to [30], the ${\hat{X}}_{g}^{i}$ and ${\hat{X}}_{h}^{j}$ have been associated when $\alpha_{gh}\leq T_{a}$. $T_{a}$ is an association threshold. Even when there are multiple ${\hat{X}}_{h}^{j}$ satisfying $\alpha_{gh}\leq T_{a}$, a smaller $\alpha_{gh}$ means a higher correlation. Thus, we fuse the data when $\alpha_{gh}$ is the minimum.

### 4.3. Multisensor Data Fusion

The convex combination fusion [30] is an optimal fusion algorithm when there is no process noise and, the local estimation of two tracks is not correlated. While the local estimation is correlated [31], the Bar-Shalom-Campo fusion algorithm [32] is better. However, due to the slow motion of targets, the effect of process noise is usually smaller. Thus, we assumed the local estimation is not uncorrelated in this paper. The two sensor estimations and covariance matrix are respectively $X^{m}$ and $P^{m}$, *m* = *i*, *j*. According to the convex combination fusion theory, the global estimation is obtained via Equation (15):

(15) $$\{\begin{array}{c} \hat{X}={\left[{\left(P^{i}\right)}^{-1}+{\left(P^{j}\right)}^{-1}\right]}^{-1}{\left(P^{i}\right)}^{-1}{\hat{X}}^{i}+{\left[{\left(P^{i}\right)}^{-1}+{\left(P^{j}\right)}^{-1}\right]}^{-1}{\left(P^{j}\right)}^{-1}{\hat{X}}^{j}\\ {\hat{P}}^{-1}={\left(P^{i}\right)}^{-1}+{\left(P^{i}\right)}^{-1} \end{array}$$

Extending to multisensor (*N* > 2) conditions, the multisensor global estimation could be derived from Equation (16):

(16) $$\{\begin{array}{c} \hat{X}=[\overset{N}{\underset{u=1}{\sum }}{\left(P^{u}\right)}^{-1}{]}^{-1}\overset{N}{\underset{u=1}{\sum }}\left({\left(P^{u}\right)}^{-1}{\hat{X}}^{u}\right)\\ {\hat{P}}^{-1}=\overset{N}{\underset{u=1}{\sum }}{\left(P^{u}\right)}^{-1} \end{array}$$

### 4.4. Feedback Algorithm Based on RFS Theory

**Figure 5:**
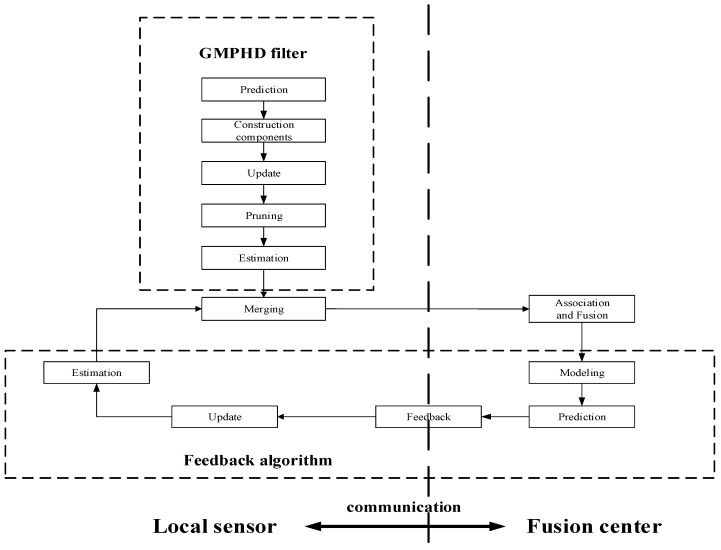
Structure of feedback algorithm.

Since GMPHD is statistically unbiased, it is possible to bias GMPHD by feeding other target information to GMPHD. We thought that the method of independent implement of feedback algorithm and GMPHD was a good way to avoid bias problem. As shown in Figure 5, at time *k* − 1, global estimations were calculated by fusion algorithm, and then modeled as a RFS. Based on the feedback target information, the state of target can be predicted for time *k*. After prediction, the prediction information will be fed back to local sensors. After that, at time *k*, we referred to the RFS theory to make a feedback estimate. Finally, the estimation of the local sensor is obtained by fusing the estimation of feedback with estimation of GMPHD filter.

This paper proposes a feedback algorithm that offered two advantages. The feedback algorithm could improve the detection ability of local sensor without biasing GMPHD filter. On the other hand, we have noted that feedback algorithm might expand the error estimation effect of GMPHD filter.

The details of the feedback algorithm are described as follows:

(1) Modeling. According to RFS theory, the feedback is modeled as a RFS:
  (17) $$\Psi_{F,k-1|k-1}=\left\{{\hat{x}}_{k-1|k-1,1},{\hat{x}}_{k-1|k-1,2},\cdots ,{\hat{x}}_{k-1|k-1,J_{F,k-1}}\right\}$$
  where $J_{F,k-1}$ is the number of global estimations, $\hat{x}$ is the state of global estimation at time *k* − 1.
(2) Prediction. Based on the transfer matrix $F_{k|k-1}$ and process noise *Q*, the target state and covariance are predicted via Equations (18) and (19):
  (18) $${\tilde{x}}_{k|k-1,j}=F_{k|k-1}{\hat{x}}_{k-1|k-1,j}$$
  (19) $$P_{F,k|k-1,j}=Q+F_{k|k-1}P_{F,k-1|k-1,j}F_{k|k-1}^{T}$$
(3) Feedback. The prediction state and covariance are fed back to the local sensors at time *k* − 1.
(4) Update. By Equations (20)–(22), we could update the target state and covariance at time *k*:
  (20) $$K_{F,k,j}=P_{F,k|k-1,j}H_{k}^{T}{\left(H_{k}P_{F,k|k-1,j}H_{k}^{T}+R_{k}\right)}^{-1}$$
  (21) $${\hat{x}}_{F,k,j}={\tilde{x}}_{F,k|k-1,j}+K_{F,k,j}\left(Z_{k,i}-H_{k}{\tilde{x}}_{F,k|k-1,j}\right)$$
  (22) $$P_{F,k,j}=\left(I-K_{F,k,j}H_{k}\right)P_{F,k|k-1,j}$$
(5) Estimation. At time *k* − 1, assuming the intensity of the feedback target is a Gaussian mixture form:
  (23) $$v_{F,k-1}\left(x\right)=\overset{J_{F,k-1}}{\underset{i=1}{\sum }}\omega_{F,k-1}^{\left(i\right)}N\left(x;m_{F,k-1}^{\left(i\right)},P_{F,k-1}^{\left(i\right)}\right)$$
  where, $N\left(•;m_{F,k-1},P_{F,k-1}\right)$ denotes a Gaussian density with mean $m_{F,k-1}$ and covariance $P_{F,k-1}$. The $\omega_{F,k-1}$ is the weight of the Gaussian density. Thus, the predicted intensity is a Gaussian mixture of form:
  (24) $${\tilde{v}}_{F,k|k-1}\left(x\right)=\overset{J_{F,k-1}}{\underset{i=1}{\sum }}\omega_{F,k|k-1}^{\left(i\right)}N\left(x;{\tilde{m}}_{F,k|k-1}^{\left(i\right)},{\tilde{P}}_{F,k|k-1}^{\left(i\right)}\right)$$
  Then, at time *k*, the intensity of measured target is a Gaussian mixture of form:
  (25) $${\bar{v}}_{F,k}\left(z\right)=\overset{J_{F,k-1}}{\underset{i=1}{\sum }}\omega_{F,k}^{\left(i\right)}N\left(z;{\tilde{m}}_{F,k|k-1}^{\left(i\right)},{\tilde{P}}_{F,k|k-1}^{\left(i\right)}\right)$$
  When the ratio of intensity of measured target to target birth intensity exceeds the feedback threshold *T_F_*, the target state will be extracted. In general, we recommend that the correlation threshold be the same as the merge threshold. All extracted targets are the feedback estimation of feedback algorithm.
  (26) $${\hat{X}}_{F,k}=\{\begin{array}{c} \left[{\hat{X}}_{F,k},z\right],\frac{{\bar{v}}_{F,k-1}\left(z\right)}{\gamma_{k}\left(z\right)}>T_{F}\\ {\hat{X}}_{F,k},else \end{array}$$
(6) Merging. At time *k*, the GMPHD filter estimations ${\hat{X}}_{k}$ and feedback estimations ${\hat{X}}_{F,k}$ are merged as the local estimations via Equations (9)–(13).

## 5. Simulation

In this section, there are four examples. All sensors could detect the targets except Example C. Considering a two-dimensional scenario, the number of measurements (contains targets and clutters) is time-varying and unknown over the surveillance region [−1000, 1000]×[−1000, 1000] (in m). At time *k*, each measurement contains location $\left(p_{x,k},p_{y,k}\right)$ and velocity $\left(v_{x,k},v_{y,k}\right)$, and is represented by $x_{k}={\left[p_{x,k},p_{y,k},v_{x,k},v_{y,k}\right]}^{T}$$x_{k}={\left[p_{x,k},p_{y,k},v_{x,k},v_{y,k}\right]}^{T}$. Each target has survival probability $P_{S,k}=0.99$ and follows the linear Gaussian Model. The transfer model *F_k_* and process noise *Q_k_* are represented as follows:

$$F_{k}=\left[\begin{array}{cc} I_{2} & \Delta I_{2}\\ 0_{2} & I_{2} \end{array}\right],Q_{k}=\sigma_{v}^{2}\left[\begin{array}{cc} \frac{\Delta^{4}}{4}I_{2} & \frac{\Delta^{2}}{2}I_{2}\\ \frac{\Delta^{3}}{2}I_{2} & \Delta^{2}I_{2} \end{array}\right]$$

where $I_{n}$ and $0_{n}$ are n×n identity and zero matrices, Δ=1s is the sampling period, and $\sigma_{v}=5\left(m/s^{2}\right)$. $H=\left[I_{2},0_{2}\right]$ is the observation model and observation noise is $R_{k}=\sigma_{\varepsilon }^{2}I_{2}$, $\sigma_{\varepsilon }=10\left(m\right)$.

**Figure 6:**
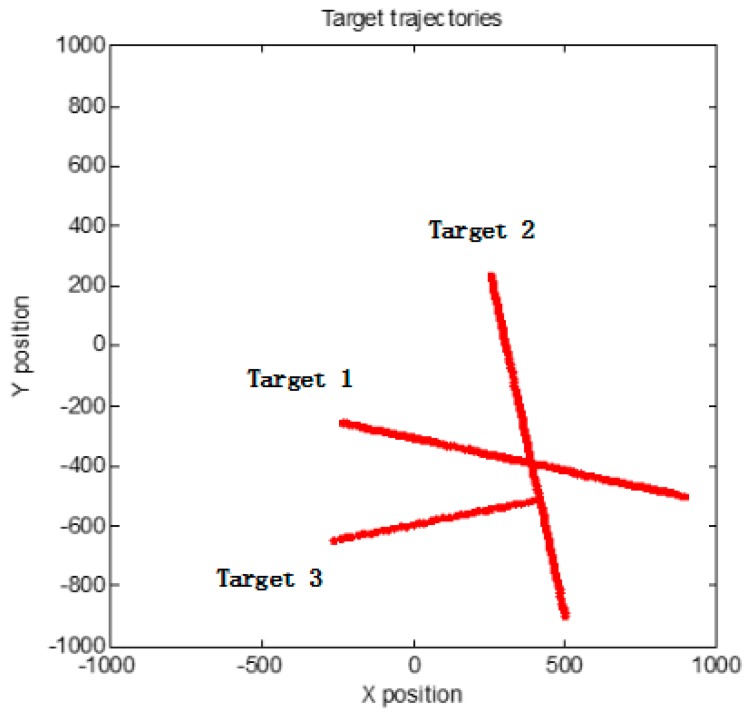
Targets trajectories.

There are three targets and clutters (less than 50) over the surveillance region. Target 1 and target 2 are born at time *k* = 0, the target 3 is spawned by target 2 at time *k* = 66. All the targets are straight uniform motion as shown in Figure 6. The number of clutter varies randomly with time.

The intensity of birth and spawn target are represented by $\gamma_{k}\left(x\right)=0.1\overset{J_{\gamma ,k}}{\underset{i=1}{\sum }}N\left(x;x,P_{\gamma }\right)$ and $\beta_{k|k-1}\left(x|Ϛ\right)=0.05N\left(x;Ϛ,Q_{\beta }\right)$, where the Ϛ is the previous state, $P_{\gamma }=diag\left({\left[100,\;100,\;25,\;25\right]}^{T}\right)$, $Q_{\beta }=diag\left({\left[100,\;100,\;400,\;400\right]}^{T}\right)$. The intensity of clutter follows uniform distribution.

In addition, the GMPHD filter parameters with detecting threshold $T_{\omega }=0.5$, merging threshold U = 4, maximum allowable number of Gaussian terms $J_{max}=100$. The feedback threshold is $T_{F}=0.5$, the association threshold is $T_{a}=4$.

The OSPA [33] is a good index for evaluating the performance of MTT algorithms. Therefore, the statistical characteristics of OSPA was used to evaluate the performance of the algorithm in this paper. The OSPA order p=1 and truncation distance c=200. We carried out 100 Monte Carlo simulations, each with 100 steps.

### 5.1. Example A. The Tracking Performance at Different Detection Probabilities

**Figure 7:**
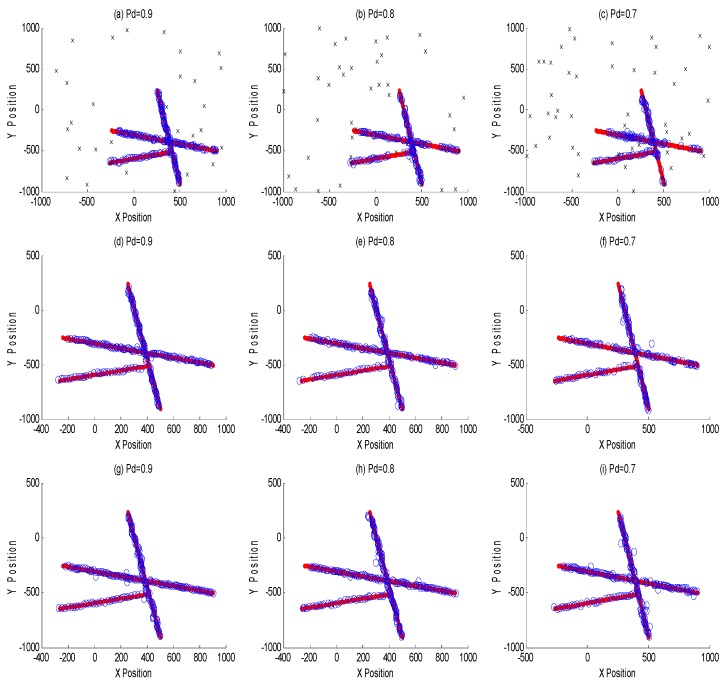
Multisensor tracking results at different detection probabilities. (**a**–**c**) are classical GMPHD filter tracking results at different detection probabilities; (**d**–**f**) are two sensors global estimations by MDC-MTF algorithm; (**g**–**i**) are three sensors global estimations by MDC-MTF algorithm.

**Figure 8:**
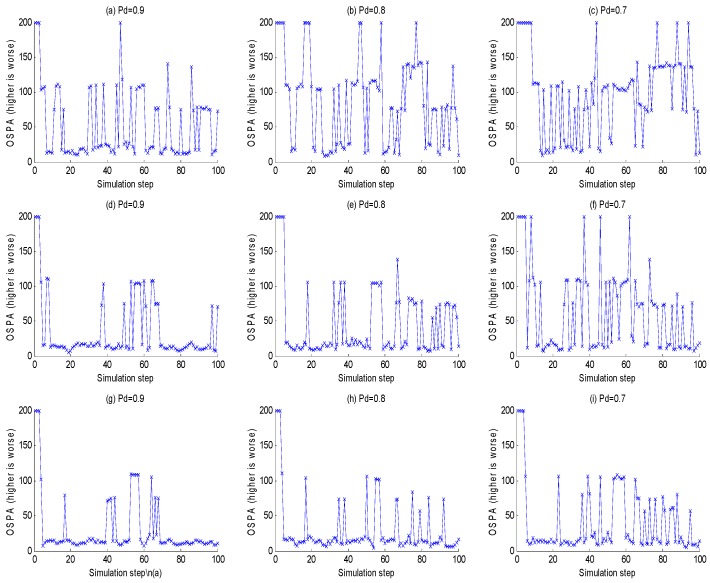
The OSPA of multisensor data fusion. (**a**–**c**) are the OSPA of GMPHD filter tracking results at different detection probabilities; (**d**–**f**) are the OSPA of two sensors global estimations by the MDC-MTF algorithm; (**g**–**i**) are the OSPA of three sensors global estimations by MDC-MTF algorithm.

**Figure 9:**
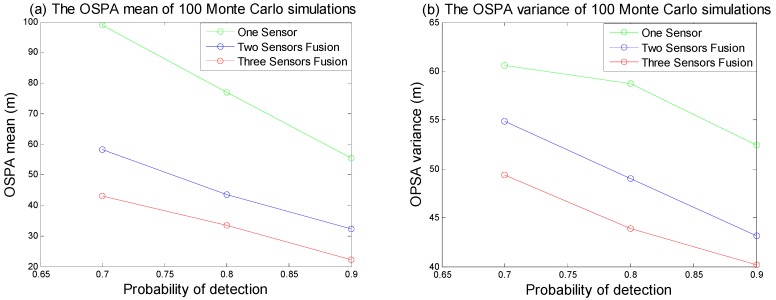
The OSPA statistical nature of Monte Carlo simulations. (**a**) is the OSPA mean of 100 times Monte Carlo simulations; (**b**) is the OSPA variance of 100 times Monte Carlo simulations.

The purpose of this example was to evaluate the performance between this tracking framework and GMPHD filter at different detection probabilities (0.9, 0.8 and 0.7). Figure 7a–c are GMPHD filter tracking results at different detection probabilities; Figure 7d–f are the global estimation based on two sensor fusion; Figure 7g–i are the global estimation based on three sensor fusion. Their corresponding OSPA results are shown in the Figure 8. The red line is the real track of the target; the blue ‘o’ is the algorithm estimation; the black ‘x’ is clutters. In order to test the robustness of this algorithm, this paper performed a Monte Carlo simulation, and calculated the OSPA statistical natures as shown in the Figure 9.

From Figure 7, Figure 8 and Figure 9, it is obvious that this algorithm has a better performance than GMPHD filter when the detection probability is low. When detection probability is 0.7, the mean of OSPA could decrease almost by 40% for two sensors fusion and 55% for three sensors fusion.

### 5.2. Example B. Feedback/No Feedback Effect

**Figure 10:**
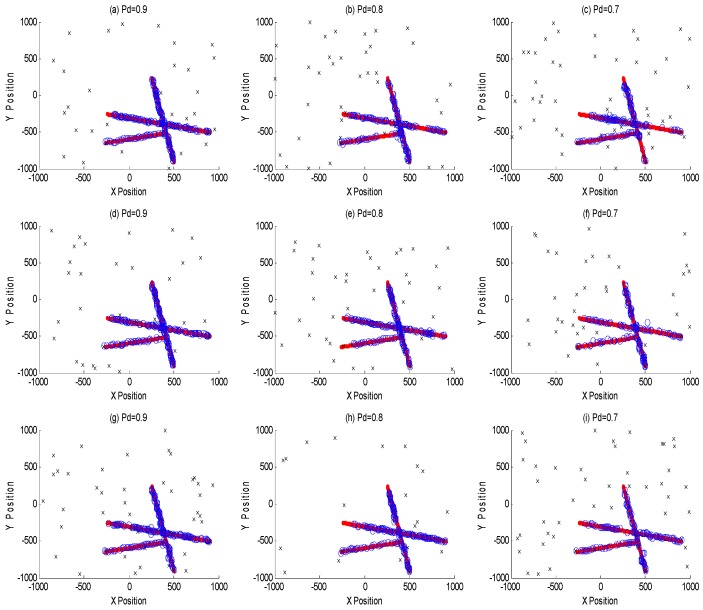
Feedback/ no feedback tracking results. (**a**–**c**) are no feedback results; (**d**–**f**) are local sensors tracking results after feeding back two sensors fusion results; (**g**–**i**) are local sensors tracking results after feeding back three sensors fusion results.

**Figure 11:**
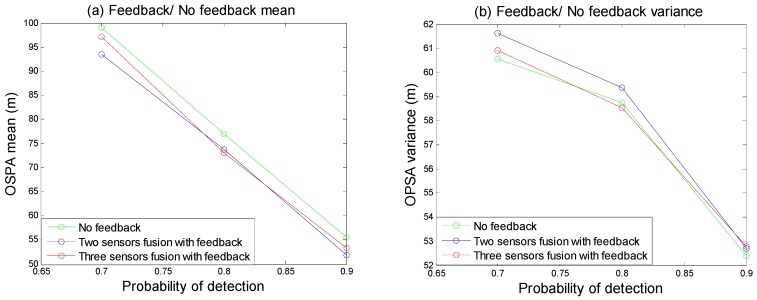
The OSPA of feedback/ no feedback. (**a**) is the OSPA mean of feedback or no feedback; (**b**) is the OSPA variance of feedback or no feedback.

In this example, the performance of feedback algorithm was evaluated. As the Figure 10 shown, the local sensor tracking results with feedback and no feedback. Figure 10a–c are no feedback results; Figure 10d–f are the local sensor tracking results in the two sensor fusion structure; Figure 10g–i are the local sensor tracking results in the three sensor fusion structure. The OSPA statistical nature of the Monte Carlo simulation is shown in the Figure 11. We could see the multitarget tracking performance of the local GMPHD filter with feedback algorithm is better than without feedback algorithm. That is because feedback algorithm could help GMPHD filter track those targets with no predicted information. Meanwhile, in Figure 11a, when the detection probability is low, the red line is higher than the blue line, this indicates the feedback algorithm may expand the impact of estimation error.

### 5.3. Example C. Simulation of a Target is in Acoustic Shadow Zone

**Figure 12:**
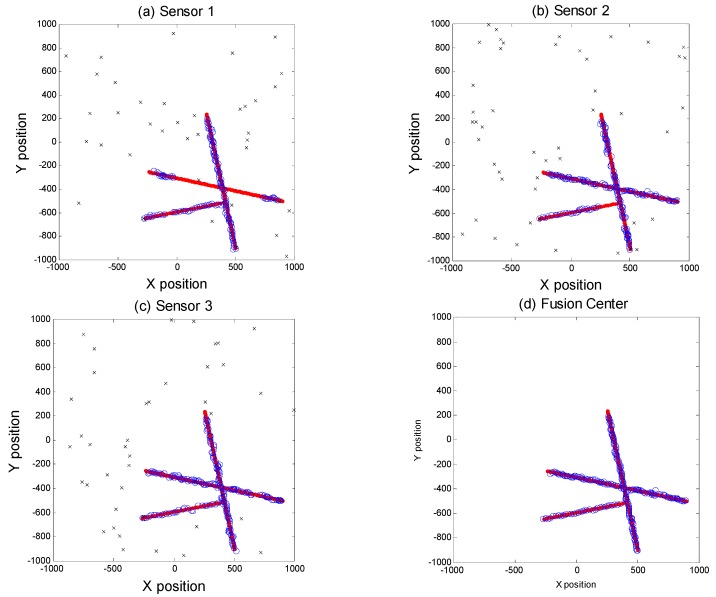
Tracking simulation of the target in acoustic shadow zone.

Considering that a wrong sensor location (e.g., an acoustic shadow zone) may make the sensor unable to detect a target, we performed a simulation based on three sensor data fusion, where the detection probability was 0.9. In this simulation, sensor 1 failed to detect the target 1 from step = 20 to step = 80. Thus, from step = 20 to step = 80, the measurement data of the target did not exist in sensor 1. As shown in Figure 12, Figure 12a–c are the local sensor tracking results, Figure 12d is the global estimation, respectively. In Figure 12, we could see the MDC-MTF algorithm can track the target in an acoustic shadow zone.

### 5.4. Example D. Analysis of the Influence of Some Important Parameters on Performance

**Figure 13:**
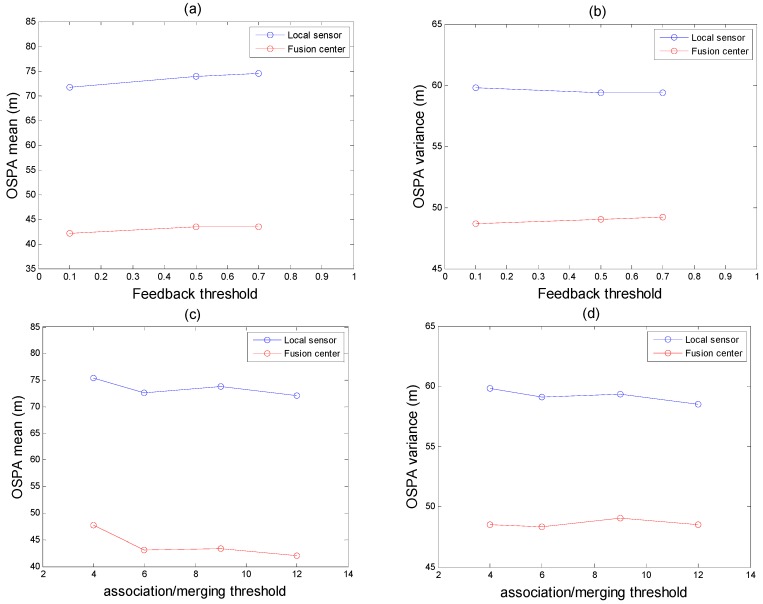
Simulation analysis of the effect of threshold on algorithm performance.

As some thresholds are important to the performance of the tracking algorithm, we will analyze the influence of the feedback threshold, merging and correlation threshold on the performance of the algorithm when the probability of detection is 0.8. As shown in Figure 13a,b are the statistics of the OSPA with different feedback thresholds, (c) and (d) are the statistics of the OSPA with different association thresholds. Figure 13 illustrated two issues: (1) A small feedback threshold means greater tolerance for measured error. However, a small feedback threshold also means the risk of clutter or error estimation increases, though feedback threshold could improve MTT tracking performance. (2) The essence of association threshold based on Mahalanobis distance is the correlation of data sets. In this paper, within a certain range, a lager threshold can improve MTT performance.

## 6. Conclusions

In this paper, a maximum-detection capability multitarget track fusion (MDC-MTF) algorithm was proposed, which contains a maximum detection capability fusion strategy, data association, multisensor data fusion and a novel feedback algorithm based on RFS theory. In the distributed sensor network, considering the complexity of computation and the sonar working environment, the GMPHD filter was selected to track local sensors. To deal with the problem that GMPHD was designed for high SNR, we associated and fused multisensor data. Moreover, this algorithm also successfully solved the problem of target tracking in acoustic shadow zones. Monte Carlo simulations have proved this algorithm’s performance. Firstly, when detection probability is 0.7, the OSPA mean of two sensors fusion could be decreased almost by 40% with GMPHD, and three sensor fusion could be decreased by almost 55%. Secondly, the feedback algorithm could improve the detection ability of local sensors without biasing the GMPHD filter. On the other hand, we have noted that feedback algorithm might expand the error estimation effect of the GMPHD filter. Thirdly, by fusing multisensor data, the MDC-MTF algorithm could track targets which were in the acoustic shadow zones of a sonar sensor.

## Figures and Tables

**Table 1:** Performance comparison of track correlation algorithm for distributed multisensor systems.

Name	Computing Time (Second)	Communication Burden	Correct Correlation Probability (Medium Target Density)	Correct Correlation Probability (High Target Density)
NN	48	low	0.6449	0.4284
k-NN	307	low	0.8922	0.7526
MK-NN	291	low	0.8956	0.7694
WTA	47	medium	0.7315	0.4755
m-WTA	138	high	0.7384	0.4901
independent-STCC	470	medium	0.9065	0.7735
dependent-STCC	1406	high	0.8294	0.7009
independent-BTC	284	medium	0.9319	0.8067
dependent-BTC	818	high	0.9143	0.7958
FSTCC	352	medium	0.9218	0.7786
